# The Impact of Public Health Insurance on Household Credit Availability in Rural China: Evidence from NRCMS

**DOI:** 10.3390/ijerph17186595

**Published:** 2020-09-10

**Authors:** Qing Yang, Qing Xu, Yufeng Lu, Jin Liu

**Affiliations:** 1 School of Public Economics and Administration, Shanghai University of Finance and Economics, 777 Guoding Rd., Shanghai 200433, China; yangqing@163.shufe.edu.cn; 2 Research Institute for Agriculture, Farmer and Rural Society in China, Shanghai University of Finance and Economics, 777 Guoding Rd., Shanghai 200433, China; xu.qing@mail.shufe.edu.cn; 3 Institute of Finance & Economics, Shanghai University of Finance and Economics, 777 Guoding Rd., Shanghai 200433, China; luyufeng421@163.com

**Keywords:** New Rural Cooperative Medical Scheme (NRCMS), public health insurance, credit availability, China, I18: G50, O16

## Abstract

A large body of literature has shown that the burden of healthcare can push individuals and households into the burden of medical care and income loss. This makes it difficult for rural or low-income households to obtain and use safe and affordable formal credit services. In 2003, China’s government implemented a new rural public health insurance, which was called the New Rural Cooperative Medical Scheme (NRCMS). This study provides evidence of the impact of NRCMS on household credit availability using the China Family Panel Studies (CFPS) for 2010. The tobit regression approach and mediator model are used. The results show that, as a public health insurance system sustained by the participation of government investment, the NRCMS provides good “collateral” and significantly enhances rural households’ formal credit availability level. Furthermore, this positive effect is mainly reflected in the economic effect of NRCMS. Our results are robust to alternative statistical methods. Our findings suggest that expanding access, fulfilling the NRCMS mortgage function, and providing more financial services for rural households would have big benefits with regard to easing credit constraints for rural residents.

## 1. Introduction

Farmers or rural households, which represent the main part of the rural economy, normally face capital constraints when making production investment decisions, and a good rural credit environment can change farmers’ initial endowment, which can make an important contribution to expanding production scale and increasing agricultural investment and income [1]. Agricultural production is vulnerable to climate change and pests. It is naturally risky, and farmers’ income lacks stability, especially in poor harvest years when they need to use loans to smooth consumption [2]. However, due to the financial environment and regulatory arrangements in rural China, there is a lack of effective collateral for rural households and insufficient credit supply from formal financial institutions. Most serious bank and insurance exclusions, therefore, occur in rural areas [3], and poorer farmers or households have a lower probability of entering the informal credit market [4]. In fact, Li et al. showed that around 54.9% of the 918 respondents in South China were credit-constrained in terms of formal finance [5].

As we know, one of the worst shocks to individuals or households is serious illness, which introduces the burden of medical cost and lowers income [6]. The survey data of the Poverty Alleviation Office of the State council showed that 42% of seven million poor peasants were poor because of illness at the end of 2015 in China (Sohu News Network: Multidimensional Elimination of “poverty due to illness”, 14 January 2016(http://mt.sohu.com/20160114/n434484593.shtml)). Poverty due to illness is one of the root causes of poverty in rural China, and 51.63% of poor people attribute their poverty to the illness of household members in rural China [7]. Importantly, health shocks and major diseases, in particular, can impact rural households’ savings not only in the short term, but also in the long term [8].

To reduce farmers’ or rural families’ health risks and the burden of medical costs, the Chinese government is committed to the establishment and improvement of the rural public healthcare system. However, the level of rural public medical security has remained relatively low for a long time due to the limited financial capacity and systematic constraints. In 2003, the State Council of the People’s Republic of China, therefore, implemented the New Rural Cooperative Medical Scheme (NRCMS) for the rural population by registering capital in public health insurance. Although the implementation responsibilities were decentralized to the county-level local governments, this voluntary and heavily subsidized scheme has seen extremely rapid growth of coverage. By the end of 2013, over 0.8 billion rural people were enrolled in NRCMS, with an average of RMB 371 per capita and 1.9 billion beneficiaries. The government’s financial support then continued to increase, with the per capita subsidy standard for the NRCMS equal to RMB 380, accounting for 76% of the total insurance premium in 2015.

In this paper, we estimate the impact of NRCMS on rural households’ formal credit availability using the data from the China Family Panels Survey (CFPS) for 2010. Our estimates show that NRCMS had a significant positive effect on improving rural households’ formal credit availability level because NRCMS, as an important source of rural public health insurance, can help individuals or households reduce medical costs and provide good “collateral”.

Past studies investigated the impact of NRCMS on access to care [9,10,11,12] and on health medical cost [13], as well as emphasized the role of nutrition or consumption [14], labor supply, or mobility [15,16,17,18]. For example, on the basis of 8273 people aged 45 or above from the China Health and Retirement Longitudinal Study (CHARLS) conducted in 2011 and 2013, Liu et al. found that NRCMS significantly reduced the elderly’s nonfarm labor force participation willingness and labor time [15]. Furthermore, Lai et al. investigated the distribution of benefits under the NRCMS across economic groups [19]. However, these contributions did not sufficiently acknowledge the impact of NRCMS on capital factors, and none looked at the effect of NRCMS on formal credit availability. Our work can fill this gap in the empirical literature, which is important from a policy perspective. In addition, this paper clarifies the effect of NRCMS on rural households’ credit availability, and it provides a new possible path for China or other developing countries to push farmers or rural households into a wide range of financial services.

The remainder of the paper is organized as follows: Section 2 describes the effect of NRCMS on credit availability in China, which is as an alternative theoretical framework. Section 3 describes the materials and methods, and Section 4 reports the results. Section 5 discusses the results. We conclude the study in Section 6.

## 2. The Effect of NRCMS on Credit Availability in China

One of the reasons for the exclusion of finance lies in the information asymmetry between the parties involved in the transaction in rural areas, such as adverse selection and moral hazard problems [3,20]. Credit institutions identify the risks of rural households when lending, and public health or other health insurance, as an effective tool to identify health risk information, can be valued by credit institutions. For example, some microfinance institutions in India also provide health insurance to borrowers or their spouses when issuing microloans [21]. This research found that compulsory health insurance not only improved the health of the borrower’s family, but also reduced the possibility that the borrower cannot repay the loan due to health risks, while it was more likely for men to use the health insurance acquired through their spouse’s loan than vice versa. Brevoort et al. also pointed out that the direct or indirect economic benefits of medical insurance for families can help improve the family’s credit qualifications [22]. It can be seen that the health and economic effects of NRCMS, as a source of public medical insurance, may also have an impact on the rural households’ credit availability, as explored below.

### 2.1. Health Effects

Health, when defined as an important “ability” [23], appears as the basis of all human activity. Many previous studies confirmed the impact of health on rural households’ economic behavior and highlighted other potential benefits. Previous studies showed that health significantly impacts labor efficiency, labor participation [24,25,26,27], and even household asset holdings [28]. Thus, health status undoubtedly affects the solvency of rural households’ families. In the current rural formal credit market, peasant households generally lack collateral, and the only exception is the small number of wealthy peasant households who can obtain large amounts of credit. Microfinance is based on farmers’ credit qualifications, which require no collateral. The amount is small and generally does not exceed RMB 50,000. The sample amount of formal credit in this paper not exceeding RMB 50,000 RMB accounts for 93.2% of all formal credit samples. Furthermore, the sample proportions of RMB 0–10,000 (inclusive), RMB 10,000–20,000, RMB 20,000–30,000, and RMB 40,000–50,000 RMB are 44.8%, 24.5%, 16.6%, 3.7%, and 3.6%, respectively. Most farmers currently mainly borrow through microcredit. The microfinance promoted by rural credit institutions, therefore, focuses to a greater extent on rural households’ credit qualifications. The current income and the property of rural households act as hard credit conditions. Currently, rural credit cooperatives and agricultural and postal saving banks generally take the health status of peasant households into account. Some loan officers regard the ownership of medical insurance such as NRCMS as a means of identification when distinguishing the family’s ability to cope with the impact of health risks. In fact, health describes the future solvency of rural households to some extent, which means it plays a signaling role in the credit process. NRCMS can improve rural households’ credit availability level through health effects.

### 2.2. Economic Effects

Credit default in peasant households is mainly caused by passive default, especially accidental passive default as a result of being overburdened (including serious illness and other shocks). Medical risk, therefore, provides an important incentive for rural households to default on credit. The imperfection of rural financial markets, the insufficient supply willingness of rural formal credit institutions, and even the exclusion of supply has produced a “reluctance to lend, prudence when lending” mentality, which has produced a low availability of formal credit for rural households and prominent illness-related poverty-stricken regions. The Chinese government directly invested in medical insurance by establishing the NRCMS with the aim of addressing this form of poverty in peasant households and reducing the economic and medical burden on rural households. In recent years, it has continuously increased financial investment, improved the level of compensation, expanded the scope of compensation for diseases and medicines, and promoted the implementation of the major illness insurance system.

Due to the government’s financial endorsement, a safety net was established for NRCMS participators to reduce their risk of defaulting due to medical burden or income loss as a result of illness. When governments intervene in the health insurance market and compensate for the insufficient supply of the financial system in rural areas, the risk of default decreases and rural households’ credit access can increase. Therefore, NRCMS can be regarded as a kind of credit collateral endorsed by the government, which is helpful to improve households’ credit availability and ease credit constraints in rural China.

## 3. Materials and Methods

### 3.1. Data

This paper uses information taken from the baseline data of the 2010 China Family Panel Studies (CFPS). CFPS is a nationally representative, annual longitudinal survey of Chinese communities, families, and individuals launched in 2010 by the Institute of Social Science Survey (ISSS) of Peking University, China. The CFPS is designed to collect individual-, family-, and community-level longitudinal data in contemporary China. The studies focus on the economic, as well as the noneconomic, wellbeing of the Chinese population, with a wealth of information covering topics such as economic activities, education outcomes, family dynamics and relationships, migration, and health. The CFPS is funded by the Chinese government through Peking University. The CFPS promises to provide the most comprehensive and highest-quality survey data on contemporary China to the academic community. More details are offered on their website (http://www.isss.pku.edu.cn/cfps/en/index.htm).

In 2010, the CFPS baseline survey covered 25 provinces/municipalities/autonomous regions with a sample size of 14,798 households, including all family members.

Most rural families participate in the NRCMS, although a small number of family members also use other medical insurance, such as urban workers’ medical insurance and supplementary medical insurance. In order to better test the impact of the NRCMS on rural households’ formal credit availability, this paper excluded urban samples that lack key variables, along with samples that include participation in other sources of medical insurance. The final effective sample contained 6020 households.

### 3.2. Variables

#### 3.2.1. Variable of Credit Availability

This paper measures households’ credit availability by referring to “whether or not” and “how much”, instituting two indicators of credit availability and credit quota: (1) the value is 1 if the household gets a loan, and 0 in all other instances; (2) the credit quota is the amount in loans that households receive. The formal credit mentioned in this paper includes both bank and credit cooperative loans.

#### 3.2.2. Variables of the NRCMS

In applying the CFPS questionnaire, this paper used two variables to measure participation in the NRCMS. Participation in the NRCMS was taken as a binary variable with 1 indicating yes, and 0 indicating all other instances. This is because formal credit is mainly applied in the name of hosts of households, which means that the household host is the decision-maker who is legally directly responsible for the household’s credit behavior. Secondly, the householder’s participation or nonparticipation conveys the information of the family’s risk type and repayment ability to some extent. When more family members participate, there is a greater cushioning effect on the impact of family health risk. This can then reduce the credit risk created by health shocks and improve the level of formal credit availability.

#### 3.2.3. Intermediate Variables

The previous theoretical mechanism established that the NRCMS may affect farmers’ availability through health and economic effects. The paper’s intermediate variable of health effect was a self-assessment status variable, presuming that credit applicants are generally household hosts. The intermediate variable of economic effect was related to whether the family faces medical economic pressure. The CFPS questionnaire divided health conditions into “healthy, general, relatively unhealthy, unhealthy, and very unhealthy”. This paper defined both “healthy” and “general” as “healthy”, with a value of 1, with the remaining options defined as “unhealthy”, with a value of 0. In addressing the variable of whether the family is facing medical pressure, this paper used methods to express whether the family’s medical cost/annual income exceeded 20%, 40%, or 60%. A value of 1 was assigned when facing medical economic pressure, while a value of 0 was assigned in all other instances.

#### 3.2.4. Other Control Variables

Like other studies, this paper also controlled for the characteristic variables of householders in order to better understand the impact that householder characteristics have on rural households’ formal credit availability. These characteristics included age, educational years, gender, and marital and political status. In CFPS questionnaires, political status refers to whether the individual participates in the party using a binary variable with 1 representing yes and 0 representing all other instances. It should be clarified that the CFPS questionnaire, when referring to party organizations, does not just contain the Communist Party of China (CPC), but also contains eight democratic parties (The Revolutionary Committee of the Chinese Kuomintang, China Democratic League, China National Democratic Construction Association, China Association for Promoting Democracy, Chinese Peasants and Workers Democratic Party, China Zhigong Party, Jiusan Society, Taiwan Democratic Self-Government League), as well as the administrative-area people’s congresses of the People’s Republic of China, the administrative-area committee of the Chinese People’s Political Consultative Conference, and other organizations. However, rural households hardly participate in these parties or organizations except for the CPC. This paper, therefore, divided the sample into CPC and non-CPC members.

Marital status was also a binary variable, with married or cohabitation i assigned a value of 1, and unmarried, divorced, or widowed assigned a value of 0. Family characteristic variables included family net income (RMB), financial assets, gift expenditure, land assets, and real estate, and all of these variables were logarithmic in the process of empirical analysis. In the CFPS questionnaire, financial assets include bonds, cash, funds, real estate, savings, stocks and the value of existing housing. Land assets are calculated on the basis of McKinney’s [29] method, which assumes that 25% of household agricultural income comes from land and 8% comes from land income). In considering that the endowment insurance may reduce liquidity constraints, with a certain impact on credit access, the family characteristic variables also included the rate of endowment insurance. This paper also controlled for the characteristics of villages and provinces.

### 3.3. Methods

First, considering the fact that the credit availability of rural households is a typical binary variable, this paper adopted the probit model to estimate the impact of the NRCMS on the credit availability of rural households. The model was set as follows:

(1) $$P(y=1|x)=\Phi \left(\alpha_{0}+\alpha_{1}x_{1}+\sum_{i=2}^{n}\alpha_{i}x_{i}+\mu_{i}\right),$$

where *y* is the variable of credit availability, P(*y* = 1) represents the possibility of getting a loan, $\Phi \left(\cdot \right)$ is the cumulative distribution function of normal distribution, $x_{1}$ is the variable of NRCMS, and $x_{2}$, $x_{3}$ *, …,* $x_{n}$ are control variables, which include household characteristics such as age, education level, gender, marital and political status, and the square of age/100 (taking into account the nonlinear impact of age), as well as family characteristics such as financial assets, gift payments, land assets, net income, and real estate; lastly, μ is the random interference term.

Second, because the paper engaged many household samples that have zero credit, the estimations would have been inconsistent if we applied ordinary least squares (OLS) regression [30]. The tobit model can correct the deviation caused by limit variables, which enables us to incorporate samples with zero credit into the model. This paper, therefore, used the tobit model to estimate the impact of the NRCMS on rural households’ credit. The model was as follows:

(2) $$\ln(1+loan)=\beta_{0}+\beta_{1}x_{1}+\sum_{j=2}^{n}\beta_{j}x_{j}+\mu_{j},$$

where loan represents the credit amount, *x*_1_ is the NRCMS variable, *x*_2_, *x*_3_, …, *x_n_* are control variables, and *µ* is the random interference term. In addition, the credit amount “loan” in the form of a logarithmic variable renders its distribution closer to a normal distribution, which helps to meet the regression model’s hypothetical form. *ln*(1 + *loan*) is also used in logarithmic form to ensure that variables are still meaningful at the value of 0.

Third, in order to ensure the mediating role of health and economic effect in the NRCMS, this paper adopted the following mediation analysis model [31,32,33,34]:

(3) $$y=\beta_{1}x_{1}+\beta_{2}x_{2}+...+\beta_{n}x_{n}+\mu_{1},$$

(4) $$m=\beta_{1}x_{1}+\beta_{2}x_{2}+...+\beta_{n}x_{n}+\mu_{2},$$

(5) $$y=\beta_{1}x_{1}+\beta_{2}x_{2}+...+\beta_{n}x_{n}+\delta \cdot m+\mu_{3},$$

where *y* is the variable of rural households’ formal credit availability, and *m* is the intermediate variable.

With regard to the significance test of an intermediate effect, this paper drew on the research of Baron and Kenny [31] to apply the following criteria: first, the intermediate variable was regressed to the variable of NRCMS in Model (4), and the variable NRCMS was found to be significant; second, the dependent variable, i.e., the rural households’ formal credit availability, was regressed to the independent variable of NRCMS in Model (3), and the variable of NRCMS was also found to be significant; third, the dependent variable “rural households’ formal credit availability” was simultaneously regressed to the intermediate variable and the variable of NRCMS in Model (5). If the intermediate variable is significant, and the coefficient of the NRCMS gets smaller and is found to be significant, then the intermediate variable has a partial mediating effect. If the coefficients of the variables of the NRCMS decrease and show no significance, the intermediate variable has a complete mediating effect.

## 4. Empirical Results

### 4.1. Sample Descriptive Results

Table 1 shows the basic descriptive statistical characteristics of the main variables. From the view of rural households’ credit availability, the availability of formal credit was 0.114, which means that 11.4% of rural households get formal credit, and the average amount of formal credit is only RMB 2969.0 per household. When assessed from the perspective of the availability of informal credit, 31.0% of rural households can obtain informal credit, and the credit line is about RMB 5600 per household, which is almost twice the amount of formal credit. The availability and amount of informal credit are not variables, and they are, therefore, not listed in Table 1. This shows that informal credit is still the main form of credit in rural areas, and this means it is still hard to make formal credit available to rural households. With regard to NRCMS variables, the average value of participation in NRCMS was 0.869, which means that 86.9% of rural households participate in NRCMS. Meanwhile, the proportion of family participation in the NRCMS was 0.820, which confirms that 82.0% of members from rural families participate in the NRCMS.

The average householder was around 50 years of age and was basically educated to a primary school level (4.7 years); 7.6% of respondents were party members, 89.5% were married, 80.6% were male, and 78.7% enjoyed good health. The average family had four members and each household had an average net income of about RMB 23,000, indicating that the rural income level is still comparatively low. Household financial assets were about RMB 5500, including bonds, cash, savings, and stocks. When calculated on the basis of a four-person household, the average assets represent less than RMB 1400, including both cash and savings. Households’ real estate and land assets represent nearly RMB 90,000 and RMB 30,000, respectively. It appears that the level of rural real estate and land assets is not low, but they are far less liquid than urban assets because rural land ownership is collective, which means it is more secure in these areas. Family gift expenditure was about 1600 RMB, which is one-quarter of the family financial assets, indicating that the cost of maintaining social relations in these areas also increases. The participation rate of family endowment insurance was only 7% because a pilot implementation of the new rural endowment insurance program only began in 2009. The previous rural endowment insurance system was not perfect in many respects, as reflected in the low participation rate in rural endowment insurance. Medical pressure (only listed if the family’s medical expense/annual income exceeds 20%) was also a factor, as almost one-third (30%) of participants had a medical economic burden. Table 1 lists the relevant variables of village characteristics.

### 4.2. The Impact of NRCMS on Formal Credit Availability

Table 2 reports the regression results for the impact of NRCMS on formal credit availability in rural China. The probit model was used in the first and second columns. The explanatory variables were the availability of formal credit for rural households, and the core explanatory variables of NRCMS were “whether to participate in the NRCMS” and the participation rate. Other variables were controlled. Columns 3 and 4 used the tobit model, where the explained variable was the rural households’ formal credit amount, and the core explanatory and control variables were the same as in columns 1 and 2.

Columns 1 and 2 show that the NRCMS variables were significant at the 5% and 1% levels, respectively, confirming that the NRCMS improves the availability of formal credit for rural households. Column 1 specifically shows that participating households were more likely to obtain formal credit than households without participation. With regard to the marginal effect, the possibility of formal credit for participating households was 3% higher than for nonparticipating households. A household’s application for formal credit is normally decided by the host, and the decision reflects the ability to resist health risk, as well as indicates information about the family’s risk type and repayment ability to some extent. The possibility that householders participate in formal credit accordingly increases. Column 2 shows that the proportion of household participation has a significant positive effect on the increase in formal credit made available to rural households. A marginal effect perspective suggests that the probability that rural households obtained formal credit increased by 1.8% for each increase in the unit of household participation. The tobit regression results for columns 3 and 4 show that the two NRCMS variables had a significant positive impact on peasant households’ formal credit, and this impact was also shown in the probit regression.

In turning to the control variable characteristics and focusing on age, we observed that the coefficients of the square of host’s age/100 were all negative and significant at a 1% statistical level, which means that the relationship between age and credit availability is an inverted U shape. The explanation mechanism establishes that young householders had insufficient ability to pay debts because of limited income, and this lowered their formal credit availability. However, as they got older, their income increased and, thus, their repayment ability also increased. If the host was a party member, it had a significant positive effect. Because most rural party members were village branch cadres or veterans, this gave them a certain capital in social relations, making it easier for them to obtain formal credit than other nonparty hosts. The logarithmic coefficient of net household income was, however, significantly positive, and this indicates that, when income was higher, solvency was stronger, and it was more likely that formal credit was obtained. The logarithm of financial assets was significantly negative in the model, which shows that rural households’ financial assets could not improve the availability of their formal credit. When rural households had more financial assets, it was less likely they faced credit constraints. The logarithm of land assets was significantly positive at the level of 1%, which indicates that rural households’ land value positively impacted their access to loans. The gift expenditure logarithm was significantly positive, indicating that rural social capital positively affected rural households’ access to formal credit.

### 4.3. Test of Health Effect of NRCMS

The previous results comprehensively proved that the NRCMS has a significant positive impact on rural households’ access to formal credit. However, this raises questions regarding the route via which the NRCMS operates to affect rural households’ access to formal credit. This section initially asks if the NRCMS significantly improved the health status of rural households and if the health of rural households with intermediate variables significantly affected their formal credit availability level. Table 3 shows the regression results obtained for the impact of the NRCMS on formal credit availability through health.

The regression results in column 1 of Table 3 show that the coefficient of participation of the head of household was positive, indicating that the NRCMS had a certain health effect, with a marginal effect also considered. The regression results in columns 2 and 3 of Table 3 show that the influencing coefficient of householders’ health on credit availability and credit amount was significantly negative at the 1% level, indicating that the health level of householders had a significant negative effect on their credit availability. The test criteria of the intermediate effect showed that the health status of rural households with intermediate variables had no intermediate effect. This result means that the route through which the NRCMS health effect was exerted on rural households’ formal credit availability was not statistically significant.

Householders’ health status reflects future solvency to some extent and could have a significant positive effect on the level of credit availability. However, the regression results in Table 3 lead toward the opposite conclusion, as already noted. One possible explanation is that the continuous advance of industrialization and urbanization generally increases rural households’ nonagricultural income compared to their farming income. The NRCMS can improve rural households’ health and promote their nonagricultural work at the same time, which leads to lower investment in agricultural production and reduces demand for production credit. The relatively low labor efficiency of rural households with poor health means that nonfarm employers “rationally” employ none or only a few people. It is, therefore, the case that rural households with poor health can often only “stay” on the land to engage in agricultural production. However, the implicit “mortgage” function of the NRCMS increases the probability these rural households can obtain credit from financial institutions.

### 4.4. Test of Economic Effect of NRCMS

As discussed above, the economic effect of the NRCMS on credit availability can be measured using the economic burden of medical treatment. Due to the “safety net” of the NRCMS, credit risk is reduced and the possibility of lending by credit institutions increases. Hence, the way to verify the economic effect was as follows: the medical economic burden of rural households was taken as an intermediate variable to examine the economic effect of NRCMS on formal credit access using the mediation analysis model. Table 4 presents the test results for the mediating effect of household economic pressure. The medical economic burden is measured by the ratio of annual family medical expenses/family income exceeds 20%. Additionally, the conclusion is same when the ratio of annual family medical expenses/family income exceeds 40% or 60%. However, the results are not listed here due to limited space.

First, with regard to the impact of household participation rate on family medical pressure, column 1 of Table 4 shows that the coefficient of household participation was significant at the 1% level, which indicates that when the participation rate is higher, it is more likely that the household will experience medical economic pressure. In the case of the marginal effect, every percentage increase in participation would generate a 4.9% increase in the probability that rural households will experience a medical economic burden. This may be because the NRCMS, as a kind of medical insurance, could create a moral hazard. It could lead to over-diagnosing, over-prescribing, and even price increases, thereby imposing a greater medical burden on rural households.

Second, with regard to the impact of medical burden on formal credit, columns 2 and 3 of Table 4 confirm that the family medical burden significantly impacts credit availability and average credit acquisition, which indicates that the medical burden significantly stimulated rural households’ access to formal credit. When compared to the regression results of Table 2, the coefficients of the NRCMS variables were smaller and became insignificant when the family medical burden variables were added as intermediate variables. The test standard of intermediate effect established that the variable of medical economic burden had a complete mediating effect.

### 4.5. Heterogeneity Test

Furthermore, in order to verify the explanation mechanism proposed above, this paper believes that, if the NRCMS has the role of credit collateral, then the medical burden of nonparticipating families must have a weaker impact on credit access than participating families. Because the participating households are protected by the safety net of the NRCMS, their credit defaults are relatively low. Even if there is a medical burden, credit institutions will still be willing to give loans. However, due to the lack of a safety net for nonparticipating families, the risk of credit default increases significantly. When there is a medical burden, there are basically no or very few credit institutions willing to lend.

For this reason, the regression below estimates the impact of the medical burden of participating families and nonparticipating families on formal credit access. The estimated results are shown in Table 5. Columns 1–4 are the estimated results of participating rural households, in which the first two columns are the estimation results of the rural households whose participation rate is 1, and the last two columns are the estimation results of the rural households whose participation rate is <1. Columns 5–6 are the estimated results of nonparticipating rural households (participation rate = 0).

The estimation results show that, as a function of the significance of the coefficients in columns 1–6, the medical economic burden has a significant positive impact on the formal credit access of participating rural households, while it has a nonsignificant positive impact on the formal credit of nonparticipating rural households, which verifies the speculation mentioned above that the “safety net” effect of the NRCMS makes the impact of the medical economic burden on formal credit access more visible to participators. Second, judging from the coefficients in columns 1–4, compared with participating farmers with a participation rate of <1, the medical economic burden has a greater impact on participating farmers with a participation rate of 1, which means that the higher participation rate of rural households comes with a larger safety net and a lower credit default rate; thus, credit institutions will show greater willingness to lend when facing the same level of medical economic burden.

### 4.6. Robustness Test

Models (1) and (2) are likely to suffer from endogenous problems. In the first instance, the endogenous source was the common adverse selection in the insurance market. This means that the participating rural households have unobservable heterogeneity, which may affect their access to credit and result in the stochastic interference terms of the econometric equation being correlated with independent variables [35]. However, reverse causality, such as access to credit, may affect rural households’ health level (to our knowledge, no empirical analysis has yet confirmed the impact of credit availability on farmer health levels) and, thus, could affect NRCMS participation. In referring to the endogenous treatment of participation, this paper sought to use the participation ratio of village members as an instrumental variable. There were two main reasons for adopting this method.

First, the implementation of the NRCMS is based on the principle of rural households’ voluntary participation. Different rural households in the same village may, therefore, show a “group effect” in participation when they make decisions (i.e., they imitate and influence each other), but the village participation rate will not affect the sample’s credit behavior. Second, the NRCMS adopts a partial burden system in the compensation mechanism, and this is especially true of the compensation mechanism for hospitalization. The principle of “more reimbursement of primary hospitals and less reimbursement of high-level hospitals” is established, and different health governance levels (including village/town hospitals, county/city hospitals, and hospitals outside the county) are determined to have different caps, payments, starting lines, and compensation ratios. This shows that rural households’ participation in decision-making behaviors is not related to the situation of village-level medical facilities, which guarantees that instrumental variables are exogenous.

Table 6 shows the regression results of the impact of the NRCMS on rural households’ formal credit availability after the use of instrumental variables. For the estimation results, when compared to the results without instrumental variables, the coefficient of “whether the householder participates” and the proportion of households did not show a significant change, indicating that, after the endogenous problem is considered, the NRCMS still has a significant positive impact on rural households’ formal credit. The paper’s results are, therefore, quite stable.

## 5. Discussion

The government in China is actively promoting inclusive finance, with the aim of making financial services more available to rural residents, particularly low-income and poor residents. However, factors such as health or disease pressure, in addition to a lack of necessary collateral, mean that it is still difficult for rural households to access formal credit services. These conclusions can help to evaluate the policy effect of the NRCMS and improve rural households’ access to formal credit, which in turn can help to achieve rural financial inclusiveness. The promotion of inclusive finance, the increased availability of farmer financial services, and the resolution of rural households’ financial difficulties (such as medical credit constraints) are all important factors in rural China.

Our results show that, when rural households face a medical burden, the availability of formal credit is significantly improved. Two questions need to be addressed: first, why does NRCMS increase the medical burden of rural households? On the one hand, although NRCMS can reduce the price of medical services and can reduce the medical expenditure under the conditions of the same medical service demand, the decline in the price of medical services can increase the demand for medical services by participators, resulting in actual medical care expenditure not falling but rising [9,10,11,12,13]. In fact, many rural individuals or households have no money for treatment when they fall ill; thus, medical expenditure is relatively small. After the implementation of NRCMS, policy subsidies led to a decline in the proportion of out-of-pocket payments for participating rural households, which stimulated the consumption of medical services by participating rural households, leading to the actual medical expenditure increasing [9,11,36]. On the other hand, as a kind of medical insurance, the NRCMS also faces the moral hazard problem of medical institutions. The NRMCS sometimes causes he medical institutions to over-prescribe or over-diagnose, thereby increasing the cost of medical treatment of insured persons [13,36]. Second, why does medical burden cause rural households’ formal credit availability to increase significantly? As pointed out above, the system’s compensation policy ensures that rural households receive a certain proportion of reimbursement compensation when they incur a medical burden. In recent years, the government has also promoted health poverty alleviation and financial poverty alleviation projects and has sought to increase financial investment and improve reimbursement levels in order to resolve the costs and difficulties associated with medical treatment. In other words, when participating rural households need to borrow for medical treatment, the government can provide them with a “government credit” mortgage that helps to relieve their credit constraints. Some regions have, in adjusting to this reality, sought to address demand for rural households’ medical credit by introducing medical consumption loans that effectively graft credit loans and new rural cooperative funds, and this has helped to address the problems that rural households encounter when they seek to finance medical treatment. For example, Kaiyang County’s Credit Union of Guizhou Province issued nearly RMB 13 million of “KangFuTong” medical loans in 2015, and this enabled 699 poor families to access medical funds (Kaiyang County: Exploring and Promoting individual Medical Consumption Loan for Poor Farmers- Guizhou News-China Net-Donghai Information; http://jiangsu.china.com.cn/html/2016/gznews_0317/4822381.html).

Meanwhile, our results show that householders’ health status reflects future solvency to some extent, and it has a significant positive effect on the level of credit availability. However, the regression results in Table 3 lead toward the opposite conclusion, as already noted. One possible explanation is that the continuous advance of industrialization and urbanization results in rural households’ nonagricultural income being generally higher than their farming income. The NRCMS can simultaneously improve rural households’ health and promote their nonagricultural work, which leads to lower investment in agricultural production and reduces demand for production credit. The relatively low labor efficiency of rural households with poor health means that nonfarm employers “rationally” generally none or only a few people. It is, therefore, the case that rural households with poor health can often only “lock in” the land to engage in agricultural production. However, the “implicit mortgage” function of the NRCMS increases the probability that these rural households can obtain credit from financial institutions.

## 6. Conclusions

This paper provides some evidence of the impact of the NRCMS on rural households’ credit availability using the CFPS for 2010. The results show that the NRCMS has a significant positive impact on rural households’ formal credit availability and credit amount, and it also highlights that this effect is mainly exerted on rural households’ formal credit availability through an economic effect.

These conclusions show that the NRCMS is a social security system that the government directly injects with capital. In addition to improving rural households’ medical security, it also provides them with good “collateral”, which helps to increase their ability to repay credit and which improves their credit access. This provides a new perspective for the government to promote inclusive finance. It is worthy to use the “collateral” function of the NRCMS or other public health insurance policies, which aims to innovate financial services, effectively combining health insurance funds and financial institutions, thereby resolving the formal credit constraints faced by rural households. This helps to achieve the policy goal of reducing poverty and increasing farmers’ income.

This paper was constrained by data limitations; thus, it was only able to explore the short-term impact of the NRCMS on rural households’ formal credit availability. Ma et al. pointed that periodic monitoring and assessment can help to adjust such policies in a timely manner to achieve the designed aims [37]. Panel tracking data can be used to comprehensively examine the impact mechanism, and this requires us to continue to track and explore these data. In terms of use, credit can be divided into production managerial credit and life consumption credit; however, subdivision data are difficult to obtain, and this is why this paper used the total credit data. Future research should, therefore, focus on a detailed study of credit use, combining the internal mechanism with credit use and conducting more targeted empirical tests.

## Figures and Tables

**Table 1:** Basic statistical description of main variables. NRCMS, New Rural Cooperative Medical Scheme.

Variables	Definition	Mean	Standard Deviation
formloanif	Formal credit availability (yes = 1, other = 0)	0.114	0.317
formloan	Formal credit amount (RMB)	2969.386	18,471.987
ln_formloan	Ln(formloan)	0.116	1.012
nrcif_ h	Whether householders participate (yes = 1, other = 0)	0.869	0.338
nrcratio	Participation in NRCMS	0.820	0.322
age_h	Age of householder (year)	49.741	12.093
ageage_h	Square of age/100	26.204	12.61
eduy_h	Education level of householder (year)	4.734	4.168
communist_h	Political status (yes = 1, no = 0)	0.076	0.264
marriage_h	Marital status (married = 1, other = 0)	0.895	0.306
gender_h	Gender (male = 1, female = 0)	0.806	0.396
healthif_h	Health status (healthy = 1, other = 0)	0.787	0.409
familysize	Family members	4.180	1.809
famincnet	Net income (RMB)	22,615.377	43,104.962
financeasset	Financial assets (RMB)	5533.927	19,937.468
houseasset	Land assets (RMB)	91,960.245	252,164.084
landasset	Real estate properties (RMB)	29,650.396	50,522.029
gift	Gift expenditure (RMB)	1607.254	2395.182
pensionratio	Participation rate of family endowment insurance	0.070	0.227
medburdenif	Whether there is medical pressure (yes = 1, no = 0)	0.265	0.441
cindinc	Economic level (income per capita, RMB)	5435.654	3200.578
cdistance	Distance to county (km)	29.870	23.423
*N*	6020		

**Table 2:** Impacts of NRCMS on householders’ formal credit availability.

	Probit Regression		Tobit Regression	
Variable	Formloanif	Formloanif	Ln_Formloan	Ln_Formloan
nrcif_h	0.173 ** (0.075)		2.582 ** (1.122)	
nrcratio		0.100 * (0.074)		1.556 * (1.103)
age_h	0.043 *** (0.016)	0.045 *** (0.016)	0.662 *** (0.239)	0.682 *** (0.240)
ageage_h	−0.062 *** (0.017)	−0.063 *** (0.017)	−0.931 *** (0.246)	−0.952 *** (0.248)
eduy_h	0.004 (0.006)	0.005 (0.006)	0.068 (0.085)	0.071 (0.085)
communist_h	0.296 *** (0.080)	0.300 *** (0.080)	4.308 *** (1.133)	4.376 *** (1.133)
marriage_h	0.124 (0.095)	0.123 (0.095)	1.790 (1.421)	1.771 (1.422)
gender_h	0.043 (0.061)	0.041 (0.061)	0.621 (0.911)	0.590 (0.912)
familysize	0.007 (0.015)	0.007 (0.015)	0.113 (0.218)	0.114 (0.218)
ln_faminc_net	0.085 *** (0.032)	0.086 *** (0.032)	1.326 *** (0.468)	1.344 *** (0.469)
ln_financeasset	−0.033 *** (0.006)	−0.033 *** (0.006)	−0.479 *** (0.083)	−0.478 *** (0.083)
ln_houseasset	0.007 (0.009)	0.007 (0.009)	0.112 (0.126)	0.114 (0.126)
ln_landasset	0.028 *** (0.007)	0.029 *** (0.007)	0.398 *** (0.108)	0.409 *** (0.108)
ln_gift	0.043 *** (0.011)	0.043 *** (0.011)	0.659 *** (0.171)	0.664 *** (0.170)
pensionratio	0.003 (0.112)	−0.002 (0.112)	0.145 (1.670)	0.076 (1.672)
ln_cindinc	0.244 *** (0.056)	0.242 *** (0.056)	3.603 *** (0.828)	3.569 *** (0.827)
cdistance	0.004 *** (0.001)	0.004 *** (0.001)	0.053 *** (0.013)	0.052 *** (0.013)
Province	Control	Control	Control	Control
constant	−6.375 *** (0.605)	−6.333 *** (0.608)	−95.594 *** (8.675)	−95.097 *** (8.729)
N	6020	6020	6020	6020
chi2 (F)	308.900 ***	300.800 ***	24.550 ***	23.760 ***
Pseudo R^2^	0.092	0.092	0.047	0.046

Note: Steady standard errors are given in the brackets; ***, **, and * represent significance at the levels of 1%, 5%, and 10%, respectively; this also applies to all tables.

**Table 3:** Test of role of health mediators (mediation model regression).

Variable	Healthif_H	Formloanif	Ln_Formloan
healthif_h	-	−0.178 *** (0.059)	−2.635 *** (0.863)
nrcif_h	0.035 (0.058)	0.173 ** (0.075)	2.564 ** (1.123)
age_h	−0.023 *** (0.002)	0.042 *** (0.016)	0.640 *** (0.238)
ageage_h ^1^	-	−0.061 *** (0.017)	−0.928 *** (0.246)
eduy_h	0.031 *** (0.005)	0.006 (0.006)	0.092 (0.085)
communist_h	0.053 (0.077)	0.301 *** (0.080)	4.375 *** (1.131)
marriage_h	−0.074 (0.065)	0.125 (0.095)	1.796 (1.417)
gender_h	0.368 *** (0.048)	0.062 (0.062)	0.896 (0.921)
familysize	0.016 (0.012)	0.007 (0.015)	0.123 (0.219)
ln_faminc_net	0.112 *** (0.022)	0.091 *** (0.032)	1.412 *** (0.471)
ln_financeasset	0.012 ** (0.005)	−0.033 *** (0.006)	−0.475 *** (0.083)
ln_houseasset	0.022 *** (0.006)	0.008 (0.009)	0.134 (0.126)
ln_landasset	−0.011 ** (0.005)	0.027 *** (0.007)	0.389 *** (0.108)
ln_gift	0.009 (0.008)	0.043 *** (0.011)	0.663 *** (0.170)
pensionratio	0.145 (0.088)	0.000 (0.112)	0.094 (1.658)
ln_cindinc	0.062 (0.046)	0.246 *** (0.057)	3.628 *** (0.830)
cdistance	−0.001 (0.001)	0.004 *** (0.001)	0.052 *** (0.013)
Province	Control	Control	Control
constant	−0.191 (0.401)	−6.287 *** (0.607)	−94.062 *** (8.688)
*N*	6020	6020	6020
Wald chi^2^ (F)	567.700 ***	317.300 ***	24.030 ***
Pseudo *R^2^*	0.096	0.095	0.048

^1^ Owing to the linear relationship between the age of the householders and the health status, the variable of squared age/100 was not included here.

**Table 4:** Mediating effect of medical economic pressure (mediation model regression).

Variable	medburdenif	formloanif	ln_formloan
medburdenif		0.231 *** (0.055)	3.443 *** (0.793)
nrcratio	0.178 *** (0.061)	0.090 (0.075)	1.415 (1.099)
age_h	0.002 (0.011)	0.046 *** (0.016)	0.698 *** (0.239)
ageage_h	0.004 (0.011)	−0.065 *** (0.017)	−0.976 *** (0.246)
eduy_h	0.002 (0.005)	0.004 (0.006)	0.063 (0.084)
communist_h	0.036 (0.073)	0.303 *** (0.080)	4.399 *** (1.129)
marriage_h	0.271 *** (0.069)	0.114 (0.095)	1.629 (1.417)
gender_h	−0.118 ** (0.048)	0.051 (0.061)	0.735 (0.908)
familysize	0.039 *** (0.012)	0.004 (0.015)	0.072 (0.219)
ln_faminc_net	−0.542 *** (0.024)	0.126 *** (0.033)	1.924 *** (0.486)
ln_financeasset	−0.026 *** (0.005)	−0.032 *** (0.006)	−0.462 *** (0.083)
ln_houseasset	0.004 (0.006)	0.007 (0.009)	0.109 (0.126)
ln_landasset	−0.005 (0.005)	0.029 *** (0.007)	0.409 *** (0.108)
ln_gift	0.009 (0.008)	0.042 *** (0.011)	0.645 *** (0.169)
pensionratio	0.222 *** (0.081)	−0.010 (0.113)	−0.040 (1.677)
ln_cindinc	0.087 * (0.045)	0.235 *** (0.057)	3.453 *** (0.827)
cdistance	−0.001 (0.001)	0.004 *** (0.001)	0.052 *** (0.013)
Province	Control	Control	Control
constant	3.106 *** (0.464)	−6.706 *** (0.616)	−100.128 *** (8.745)
*N*	6020	6020	6020
Wald chi^2^ (F)	827.020 ***	318.210 ***	24.250 ***
Pseudo *R^2^*	0.146	0.096	0.048

**Table 5:** The impact of the medical economic burden of participators and nonparticipators on formal credit availability.

	Participators				Nonparticipators	
	Participation Rate = 1		0 < Participation Rate < 1		Participation Rate = 0	
	Formloanif	Ln_Formloan	Formloanif	Ln_Formloan	Formloanif	Ln_Formloan
medburdenif	0.208 ***	3.193 ***	0.295 **	3.959 **	0.314	4.757
	(0.065)	(0.942)	(0.120)	(1.617)	(0.219)	(3.122)
age_h	0.049 ***	0.735 ***	0.070 *	1.013 *	−0.023	−0.216
	(0.019)	(0.275)	(0.042)	(0.590)	(0.053)	(0.709)
ageage_h	−0.063 ***	−0.947 ***	−0.100 **	−1.430 **	−0.017	−0.384
	(0.019)	(0.283)	(0.044)	(0.609)	(0.057)	(0.787)
eduy_h	0.005	0.066	−0.008	−0.109	0.029	0.425
	(0.007)	(0.101)	(0.012)	(0.168)	(0.023)	(0.323)
communist_h	0.227 **	3.291 **	0.592 ***	8.066 ***	0.176	3.107
	(0.097)	(1.384)	(0.157)	(2.038)	(0.381)	(5.568)
marriage_h	0.117	1.599	−0.083	−1.045	0.313	4.938
	(0.112)	(1.667)	(0.227)	(3.166)	(0.394)	(5.691)
gender_h	0.036	0.556	0.067	0.803	0.197	2.603
	(0.075)	(1.102)	(0.125)	(1.774)	(0.230)	(3.318)
familysize	−0.003	−0.024	0.007	0.105	0.011	−0.008
	(0.018)	(0.259)	(0.034)	(0.473)	(0.065)	(0.950)
ln_faminc_net	0.136 ***	2.084 ***	0.072	0.982	0.250 **	3.823 **
	(0.039)	(0.564)	(0.075)	(1.042)	(0.120)	(1.727)
ln_financeasset	−0.037 ***	−0.541 ***	−0.018	−0.251	−0.033	−0.455
	(0.007)	(0.100)	(0.012)	(0.161)	(0.024)	(0.330)
ln_houseasset	0.005	0.079	0.023	0.363	−0.019	−0.300
	(0.010)	(0.146)	(0.021)	(0.296)	(0.029)	(0.423)
ln_landasset	0.026 ***	0.377 ***	0.032 **	0.428 *	0.040 *	0.563 *
	(0.009)	(0.132)	(0.016)	(0.226)	(0.020)	(0.289)
ln_gift	0.057 ***	0.874 ***	−0.005	−0.082	0.068 **	0.975 **
	(0.014)	(0.204)	(0.024)	(0.336)	(0.034)	(0.484)
pensionratio	0.107	1.740	−0.464	−6.552	−0.138	−2.287
	(0.129)	(1.910)	(0.288)	(4.104)	(0.381)	(5.350)
ln_cindinc	0.294 ***	4.359 ***	0.153	2.113	−0.029	−0.750
	(0.066)	(0.963)	(0.127)	(1.764)	(0.206)	(2.995)
cdistance	0.003 ***	0.045 ***	0.006 ***	0.086 ***	0.003	0.039
	(0.001)	(0.016)	(0.002)	(0.026)	(0.003)	(0.048)
Province	Control	Control	Control	Control	Control	Control
Constant	−7.441 ***	−111.326 ***	−5.280 ***	−74.300 ***	−4.368 **	−64.796 **
	(0.718)	(10.103)	(1.432)	(19.682)	(2.039)	(28.568)
*N*	4211	4211	1206	1206	603	603
Wald chi^2^ (F)	222.400	17.780	75.500	6.464	73.760	9.135
*p* > chi^2^ (*p* > F)	0.000	0.000	0.000	0.000	0.000	0.000

**Table 6:** The impact of NRCMS on rural households’ formal credit availability (using instrumental variable).

	Iv-Probit Model		Iv-Tobit Model	
Variable	Formloanif	Formloanif	Ln_Formloan	Ln_Formloan
nrcif_h	1.052 *** (0.186)		16.414 *** (3.138)	
nrcratio		1.058 *** (0.188)		16.539 *** (3.165)
age_h	0.044 *** (0.016)	0.050 *** (0.016)	0.697 *** (0.244)	0.800 *** (0.246)
ageage_h	−0.061 *** (0.016)	−0.068 *** (0.016)	−0.969 *** (0.251)	−1.070 *** (0.253)
eduy_h	0.002 (0.006)	0.003 (0.006)	0.037 (0.086)	0.050 (0.086)
communist_h	0.245 *** (0.077)	0.260 *** (0.077)	3.701 *** (1.145)	3.941 *** (1.146)
marriage_h	0.115 (0.092)	0.109 (0.092)	1.729 (1.431)	1.644 (1.436)
gender_h	0.035 (0.060)	0.011 (0.060)	0.516 (0.923)	0.142 (0.934)
familysize	0.008 (0.014)	0.012 (0.014)	0.143 (0.221)	0.197 (0.222)
ln_faminc_net	0.080 *** (0.031)	0.090 *** (0.031)	1.303 *** (0.472)	1.459 *** (0.471)
ln_financeasset	−0.031 *** (0.005)	−0.031 *** (0.005)	−0.476 *** (0.083)	−0.473 *** (0.084)
ln_houseasset	0.008 (0.008)	0.009 (0.008)	0.134 (0.128)	0.143 (0.129)
ln_landasset	0.014 * (0.007)	0.014 * (0.007)	0.208 * (0.115)	0.206 * (0.114)
ln_gift	0.030 *** (0.011)	0.030 *** (0.011)	0.478 *** (0.175)	0.482 *** (0.175)
pensionratio	0.046 (0.108)	0.050 (0.108)	0.838 (1.685)	0.905 (1.685)
ln_cindinc	0.253 *** (0.056)	0.247 *** (0.055)	3.898 *** (0.862)	3.816 *** (0.860)
cdistance	0.004 *** (0.001)	0.003 *** (0.001)	0.059 *** (0.014)	0.052 *** (0.013)
Province	Control	Control	Control	Control
constant	−6.880 *** (0.599)	−7.013 *** (0.605)	−107.582 *** (9.465)	−110.067 *** (9.657)
*N*	6020	6020	6020	6020
Wald chi^2^	380.900 ***	373.450 ***	426.360 ***	419.620 ***
Wald test of exogeneitychi^2^	24.010 ***	28.140 ***	24.760 ***	28.870 ***

## References

[1] Feder G., Lau L.J., Lin J.Y., Luo X. (1990). The relationship between credit and productivity in Chinese agriculture: A microeconomic model of disequilibrium. Am. J. Agric. Econ..

[2] Duong P.B., Izumida Y. (2002). Rural Development Finance in Vietnam: A Microeconometric analysis of household surveys. World Dev..

[3] Stiglitz J.E., Weiss A. (1981). Credit rationing in markets with imperfect information. Am. Econ. Rev..

[4] Yuan Y., Xu L. (2015). Are poor able to access the informal credit market? Evidence from rural households in China. China Econ. Rev..

[5] Li C., Lin L., Gan C. (2016). China credit constraints and rural households’ consumption expenditure. Finan. Res. Lett..

[6] Vo T., Van P.H. (2019). Can health insurance reduce household vulnerability? Evidence from Viet Nam. World Dev..

[7] Zhou G., Chen R., Chen M. (2020). Equity in health-care financing in China during the progression toward universal health coverage. China Econ. Rev..

[8] Smith J.P. (2015). Economic shocks, early life circumstances and later life outcomes: Introduction. Econ. J..

[9] Cheng L., Liu H., Shen K., Zhang Y., Zeng Y. (2015). The impact of health insurance on health outcomes and spending of the elderly: Evidence from China’s New Cooperative Medical Scheme. Health. Econ..

[10] Hou Z., Van De Poel E., Van Doorslaer Y., Yu B., Meng Q. (2013). Effects of NCMS on access to care and financial protection in China. Health Econ..

[11] Wagstaff A., Lindelow M., Jun G., Ling X., Juncheng Q. (2009). Extending health insurance to the rural population: An impact evaluation of China’s new cooperative medical scheme. J. Heal. Econ..

[12] Zhong H. (2010). Effect of patient reimbursement method on health-care utilization: Evidence from China. Health Econ..

[13] Zhou Y., Guo Y., Liu Y. (2020). Health, income and poverty: Evidence from China’s rural household survey. Int. J. Equity Heal..

[14] Bai C.-E., Wu B. (2014). Health insurance and consumption: Evidence from China’s New Cooperative Medical Scheme. J. Comp. Econ..

[15] Liu J., Lu Y., Xu Q., Yang Q. (2019). Public health insurance, non-farm labor supply, and farmers’ income: Evidence from New Rural Cooperative Medical Scheme. Int. J. Environ. Res. Public Health.

[16] Qin X., Pan J., Liu G.G. (2014). Does participating in health insurance benefit the migrant workers in China? An empirical investigation. China Econ. Rev..

[17] Shen Z., Parker M., Brown D., Fang X. (2017). Effects of public health insurance on labor supply in rural China. Ann. Glob. Health.

[18] Zhang J.H., Liu J., Xu Q. (2016). New rural cooperative medical system, land transfer and land retention. Manag. World.

[19] Lai S., Shen C., Xu Y., Yang X., Si Y., Gao J., Zhou Z., Chen G. (2018). The distribution of benefits under China’s new rural cooperative medical system: Evidence from western rural China. Int. J. Equity Health.

[20] Feder G., Onchan T., Raparla T. (1988). Collateral, guaranties and rural credit in developing countries: Evidence from Asia. Agric. Econ..

[21] Rai A., Ravi S. (2011). Do spouses make claims? Empowerment and micro-finance in India. World Dev..

[22] Brevoort K.P., Grodzicki D., Hackmann M.B. (2017). Medicaid and financial health. SSRN Electron. J..

[23] Sen A. (1999). Development as Freedom.

[24] Cai L. (2010). The relationship between health and labour force participation: Evidence from a panel data simultaneous equation model. Labour Econ..

[25] Cai L., Mavromaras K., Oguzoglu U. (2013). The effects of health status and health shocks on hours worked. Health Econ..

[26] Grossman M. (1972). On the concept of health capital and the demand for health. J. Polit. Econ..

[27] Mushkin S.J. (1962). Health as an Investment. J. Polit. Econ..

[28] Mitra S., Palmer M., Mont D., Groce N. (2015). Can households cope with health shocks in Vietnam?. Health Econ..

[29] McKinley T., Griffin K. (1993). The distribution of land in rural China. J. Peasant. Stud..

[30] Wooldridge J.M. (2003). Econometric Analysis of Cross Section and Panel Data.

[31] Baron R.M., Kenny D.A. (1986). The moderator-mediator variable distinction in social psychological research: Conceptual, strategic, and statistical considerations. J. Pers. Soc. Psychol..

[32] MacKinnon D.P., Lockwood C.M., Hoffman J.M., West S.G., Sheets V. (2002). A comparison of methods to test mediation and other intervening variable effects. Psychol. Methods.

[33] MacKinnon D.P., Luecken L.J. (2008). How and for whom? Mediation and moderation in health psychology. Health Psychol..

[34] Rucker D.D., Preacher K.J., Tormala Z.L., Petty R. (2011). Mediation analysis in social psychology: Current practices and new recommendations. Soc. Pers. Psychol. Compass.

[35] Cohen A., Siegelman P. (2010). Testing for adverse selection in insurance markets. J. Risk Insurance.

[36] Lei X., Lin W. (2009). The New Cooperative Medical Scheme in rural China: Does more coverage mean more service and better health?. Health Econ..

[37] Ma J., Xu J., Zhang Z., Wang J. (2016). New cooperative medical scheme decreased financial burden but expanded the gap of income-related inequity: Evidence from three provinces in rural China. Int. J. Equity Health.

